# Characterization of the clinical forms of chronic chikungunya: an observational cohort study in Rio de Janeiro

**DOI:** 10.1016/j.ijid.2026.109050

**Published:** 2026-10

**Authors:** Josephine Bourner, Alessandra Dantas do Nascimento, Wim Alexander Fleischmann, Jeni Stolow, Leonardo L.S. Bastos, Grazielle Viana Ramos, Patricia T. Bozza, Thiago L. Moreno Souza, Jan Felix Drexler, Jose Cerbino Neto, Fernando A. Bozza

**Affiliations:** 1 Fundação Oswaldo Cruz (Fiocruz), Rio de Janeiro, Brazil; 2 Instituto D'Or de Pesquisa e Ensino, Rio de Janeiro, Brazil; 3 University Hospitals Heidelberg, Heidelberg, Germany; 4 Tulane University, New Orleans, United States; 5 Pontifícia Universidade Católica do Rio de Janeiro, Rio de Janeiro, Brazil; 6 Laboratório de Imunofarmacologia, IOC, Fiocruz, Rio de Janeiro, Brazil; 7 Program on Surveillance in Health, Igaçu University, Nova Iguaçu, Brazil; 8 Center for Technological Development in Health (CDTS), Fiocruz, National Institute for Science and Technology on Innovation on Diseases of Neglected Populations (INCT/IDPN), Rio de Janeiro, Brazil; 9 Charité – Universitätsmedizin Berlin, Berlin, Germany; 10 Berlin Institute of Health, Berlin, Germany; 11 German Centre for Infection Research (DZIF), Berlin, Germany; 12 Nova Medical School, Lisbon, Portugal

**Keywords:** Chikungunya, Long-term complications, Risk factors, Quality of life

## Abstract

**Objectives:**

This study aimed to characterize the clinical presentation of acute and chronic chikungunya virus infection in Brazil and identify predictors of chronic chikungunya.

**Methods:**

We conducted a prospective observational cohort study in the emergency department of Hospital Quinta D’Or, Rio de Janeiro, between March 2016 and May 2017. Adults with suspected chikungunya were tested by RT-PCR for chikungunya, dengue, and Zika viruses. Patients with laboratory-confirmed chikungunya were enrolled. Clinical and demographic data were collected at baseline. At 90 days (D90), participants completed telephone follow-up assessing persistent symptoms, pain, and quality of life. Chronic disease was defined as ongoing arthralgia or myalgia at D90.

**Results:**

Among 134 PCR-confirmed cases, 100 completed follow-up and 38% reported chronic manifestations. Female sex (adjusted odds ratio [OR] 2.56, 95% confidence interval [CI] 1.02-6.25) and increasing age (adjusted OR 1.04, 95% CI 1.01-1.07) were independently associated with chronic disease. Prostration and retro-orbital pain showed trends towards association. Chronic symptoms were associated with pain interference affecting mood, work, and sleep.

**Conclusions:**

Over one-third of adults with laboratory-confirmed chikungunya experienced persistent symptoms at D90, with substantial functional impact. Early identification of patients at risk may support targeted follow-up and integration of long-term care into arboviral disease management.

## Highlights

• Chronic chikungunya disease was identified in 38% of participants.
• Female sex and older age predicted chronic symptoms.
• Chronic chikungunya pain disrupted mood, sleep, and work activities.
• Early risk identification may improve referral, pain care, and rehab.

## Background

Chikungunya is a mosquito-borne viral disease caused by the chikungunya virus (CHIKV), an alphavirus transmitted primarily by *Aedes aegypti* and *Aedes albopictus*. In recent years, chikungunya has spread rapidly across tropical and subtropical regions, driven in part by climate change, causing large outbreaks associated with acute and long-lasting morbidity and is recognized to have substantial pandemic potential [1,2]. The disease course is heterogeneous and is characterized by three distinct phases: acute, post-acute, and chronic—although not all patients experience each phase [3]. In the acute phase, patients experience abrupt onset of fever, polyarthralgia, myalgia, headache, and rash, with joint pain that may persist in the post-acute phase [4]. In the chronic phase patients may experience persistent musculoskeletal symptoms, including arthritis and arthralgia, leading to significant individual and public health burden [5,6].

Brazil has experienced sustained transmission of chikungunya virus since the first documented autochthonous cases in 2014, following its introduction into a largely immunologically naïve population [7]. Since then, chikungunya has caused recurrent and often large-scale epidemics across the country, with marked geographic heterogeneity and periodic resurgences affecting the Northeast, Southeast, and Central-West regions [8,9]. Multiple epidemic waves have been reported, reflecting complex interactions between viral lineages, vector density, population immunity, and climatic and socio-environmental factors [7,10]. As a result, chikungunya has become an established endemic arboviral disease across all states in Brazil, with ongoing unpredictable outbreaks. This trend places increasing burden on the healthcare system through rising direct and indirect costs for both patients and providers, compounded by long-term economic impacts associated with the chronic nature of the disease [9].

Beyond its acute presentation, chikungunya virus infection is frequently associated with chronic manifestations, particularly persistent arthralgia, arthritis, fatigue, and functional impairment lasting months to years after infection that may be continuous or intermittent [11,12]. Globally, longitudinal studies and systematic reviews estimate that 40-50% of patients develop chronic symptoms (from musculoskeletal symptoms to fatigue, neurological and mental health disorders) beyond 3 months, with a substantial proportion experiencing long-term reductions in quality of life [13,14]. In Brazil, a high burden of chronic musculoskeletal disease has been documented particularly among women, people with hypertension, and older adults, although reported prevalence varies widely across settings [15,16]. However, much of the existing literature relies on retrospective designs, or limited follow-up, and few prospective studies have systematically characterized both early clinical features and long-term functional outcomes using validated instruments [17]. As a result, current evidence remains insufficient to fully define the natural history and predictors of chronic chikungunya, leaving important gaps in clinical guidance and patient understanding despite the growing scale of this problem.

This study prospectively characterizes the clinical presentation of acute and chronic chikungunya virus infection in Brazil. By systematically documenting demographic characteristics, clinical signs and symptoms, and disease outcomes over time, this study seeks to improve understanding of the clinical spectrum of chikungunya in an endemic setting. These findings may inform clinical management, support differential diagnosis in areas of arboviral co-circulation, and contribute to public and international health strategies for surveillance and patient care.

## Methods

This study was approved by the Comitê de Ética em Pesquisa of Copa D’Or (CAAE no. 83369218.7.0000.5249; approval ID: 2.528.693) and was registered with the Coordenação de Pesquisa of the Instituto Nacional de Infectologia Evandro Chagas, Fundação Oswaldo Cruz (Fiocruz INI; registration ID no. 1395).

This study is reported in accordance with the Strengthening the Reporting of Observational Studies in Epidemiology (STROBE) guidelines.

### Study objectives

The aim of this study was to clinically characterize the different clinical forms of chikungunya virus infection in adults, to support differential diagnosis with other arboviral infections, and to describe the frequency of long-term manifestations associated with chikungunya and their impact on quality of life.

### Study design and setting

This was a prospective observational cohort study conducted in the emergency department of Hospital Quinta D’Or, Rio de Janeiro, Brazil. Hospital Quinta D’Or is a tertiary-care hospital. The hospital is part of a large national healthcare network and provides comprehensive secondary and tertiary medical care, including emergency services, intensive care, and specialized outpatient clinics, academic and research activities in the hospital are developed with the D´Or Institute of Research and Education (IDOR), a non-profit research Centre related to the network. The Centre covers a large catchment area of north of Rio de Janeiro, serving predominantly an insured patient population.

### Participants

Adult patients (aged ≥18 years) presenting to the emergency department of Hospital Quinta D’Or between March 2016 and May 2017 with a clinical suspicion of chikungunya (defined as: 1) Recent travel or residence in a geographic area with known Chikungunya transmission; 2) Fever upon presentation to the emergency department, 3) clinical suspicion by the attending physician) were screened for participation in the study. All potential participants were tested via RT-PCR for chikungunya, dengue, and Zika. Participants were eligible for inclusion if they had a laboratory-confirmed diagnosis of chikungunya virus infection (Supplementary material, Text S1 and Table S1). No exclusion criteria were applied.

### Data collection and follow-up

Following written informed consent, baseline data were collected at presentation to the emergency department on day 1 (D1). Data collection on a standardized case record form (CRF) included demographic characteristics, patient history, clinical signs and symptoms, presence of severe manifestations, and viral load. Data relating to ongoing chikungunya manifestations and quality of life were collected on D90.

At 90 days after enrolment, participants were followed up by telephone. Information was collected on the occurrence of auditory or visual alterations, hospitalizations since the last study visit, and the presence of arthralgia or musculoskeletal pain. Pain in the arms and legs was assessed using the Brief Pain Inventory (BPI) [18], and quality of life was evaluated using the Medical Outcomes Study Short-Form 12-Item Health Status Survey (SF-12v2) [19]. The questionnaires have been validated in Brazilian Portuguese and Portuguese, respectively. Chronic symptoms were defined as the presence of arthralgia or any muscle pain at the time of the telephone follow-up.

### Statistical analysis

Participants were included in the final analysis if they had a complete follow-up visit at D90.

Participants were retrospectively categorized into two groups according to the reported presence or absence of chronic manifestations at the D90 telephone interview. Because enrolment occurred during the acute illness rather than at a uniform interval from symptom onset, the D90 assessment did not correspond to exactly 90 days after symptom onset for all participants. Chronic chikungunya was therefore operationally defined as the presence of arthralgia or musculoskeletal pain at the D90 assessment, irrespective of whether symptoms had been continuous or intermittent since the acute infection.

The data analysis included only complete data; no missing value imputation was performed. The completeness of all study variables is summarized in Supplementary Material, Table S2.

Baseline characteristics were summarized using medians and interquartile ranges (IQR) for continuous variables and frequencies and percentages for categorical variables. Viral load was quantified from samples collected at the time of enrolment and expressed as log10 copies/mL. Viral load data were available for 83 participants and were missing for 17 participants (Supplementary material Table S2), of whom 11 were classified as having chronic manifestations at the D90 follow-up assessment. Analyses evaluating the association between viral load and chronic chikungunya were restricted to participants with available viral load measurements (complete-case analysis); participants with missing viral load data were excluded from these analyses.

Comparisons between participants with and without chronic chikungunya at D90 were performed using the chi-square test or Fisher’s exact test for categorical variables, as appropriate, and the Student’s t-test or Wilcoxon rank-sum test for continuous variables depending on distribution.

Bivariable analyses were conducted to evaluate the association between baseline characteristics and chronic chikungunya. Variables with a *P*-value <0.20 in bivariable analyses, as well as variables considered clinically relevant based on prior literature, were considered candidates for multivariable modelling.

A multivariable logistic regression model was constructed to identify independent predictors of chronic chikungunya. Adjusted odds ratios (aOR) with 95% confidence intervals (CI) are reported. Model selection was guided by clinical plausibility and statistical significance, while considering the number of outcome events to avoid model overfitting [20]. Statistical significance was defined as a two-sided *P*-value <0.05.

All analyses were performed using R version 4.5.2.

## Results

Between March 2016 and May 2017, a total of 245 patients with suspected chikungunya virus infection were enrolled in the study, of whom 134 had a chikungunya virus infection confirmed by PCR (Figure 1). At D90,100 participants were successfully contacted and interviewed by telephone to assess long-term manifestations of chikungunya. Among these, 38/100 reported chronic manifestations, whereas 62/100 did not.

**Figure 1:**
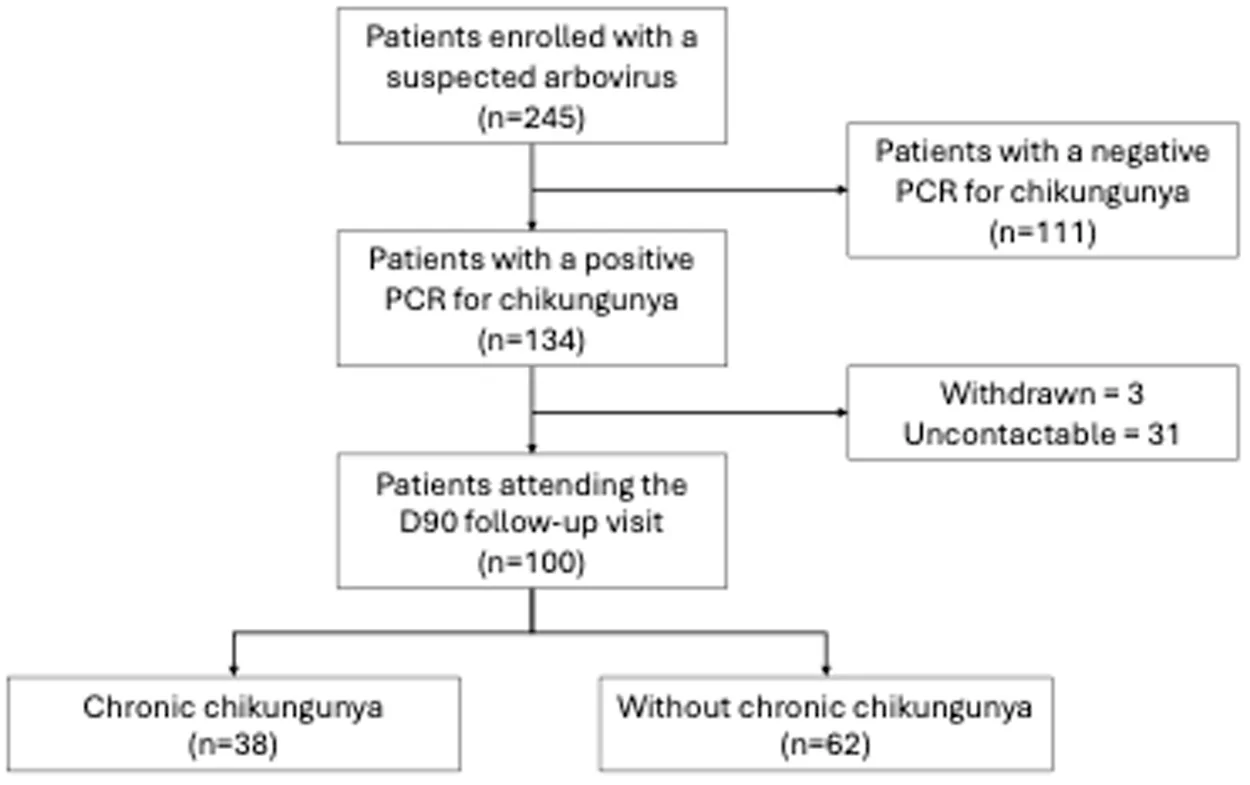
Flowchart of inclusion and follow-up of participants enrolled in the study following presentation to the emergency department.

Among the 100 participants with chikungunya who completed follow-up, 50/100 were female, and the median age of the cohort was 44 years (range: 19-87 years) (Table 1). Comorbidities were reported by 31 (31%) participants, of whom 25 (81%) had a previous diagnosis of hypertension, eight (21%) had diabetes, one (3%) had chronic obstructive pulmonary disease and one (3%) had an undefined cardiopathy. Two participants were pregnant at the time of enrolment, one of whom had chronic manifestations of chikungunya.

**Table 1:** Demographic characteristics and acute phase signs and symptoms of the 100 participants with RT-PCR confirmed chikungunya included in the final analysis.

	All participants (n = 100)	With chronic manifestations (n = 38)	Without chronic manifestations (n = 62)	*P*-value
***Demographic characteristics***				
Sex (female:male)	50:50	25:13	25:37	0.0398
Age (y), median (range)	44 (19-87)	46 (28-87)	37 (19-85)	0.0154
Pregnancy (%)	2 (2%)	1 (3%)	1 (2%)	0.6637
Comorbidities				
Hypertension (%)	25 (25%)	13 (34%)	12 (19%)	0.1534
Diabetes (%)	8 (8%)	5 (13%)	3 (5%)	0.2675
COPD (%)	1 (1%)	1 (3%)	0 (0%)	0.8038
Cardiopathy (%)	1 (1%)	0 (0%)	1 (2%)	1.0000
Multiple comorbidities	7 (7%)	5 (13%)	2 (3%)	0.1015
***Acute phase signs and symptoms***				
Time between symptom onset and presentation (d), median (range)	2 (1-7)	2 (1-5)	2 (1-7)	
Viral load (log10 copies/mL), median (range)	1.8 (−1.09 to 4.33)	1.98 (−1.1 to −4.05)	1.8 (−0.52 to −4.33)	
Fever, n (%)	98 (98%)	38 (100%)	60 (97%)	0.266
Arthralgia, n (%)	94 (94%)	37 (97%)	57 (92%)	0.269
Myalgia, n (%)	77 (77%)	30 (79%)	47 (76%)	0.719
Headache, n (%)	75 (75%)	32 (82%)	43 (69%)	0.098
Prostration, n (%)	47 (47%)	23 (61%)	24 (38%)	0.035
Retro-orbital pain, n (%)	41 (41%)	22 (58%)	19 (31%)	0.013
Rash, n (%)	35 (35%)	16 (42%)	19 (31%)	0.129
Nausea and vomiting, n (%)	31 (31%)	12 (32%)	19 (31%)	0.922
Limb oedema, n (%)	24 (24%)	9 (24%)	15 (24%)	0.954
Weakness, n (%)	20 (20%)	7 (18%)	13 (21%)	0.758
Tiredness, n (%)	4 (4%)	3 (8%)	1 (2%)	0.133
Conjunctivitis, n (%)	2 (2%)	1 (3%)	1 (2%)	0.725
Difficulty swallowing, n (%)	2 (2%)	2 (5%)	0	0.069
Arthritis, n (%)	1 (1%)	1 (2.6%)	0	0.482

Chronic chikungunya manifestations in our cohort were significantly more frequent among female participants as well as in older individuals. Although other studies have reported an association between pre-existing hypertension and chronic manifestations [16,21], this study found no evidence of hypertension—or any other evaluated comorbidity—being significantly associated with chronic chikungunya.

### Acute manifestations at presentation

The median interval between symptom onset and presentation to the emergency department was 2 days across the entire cohort. At initial presentation, the most frequently reported signs and symptoms were fever (98%), arthralgia (94%), myalgia (77%), and headache (75%) (Table 1; Supplementary material, Figure S1). Prostration (47%), retro-orbital pain (41%), rash (35%), and nausea and vomiting (31%) were also commonly reported.

Prostration (inability to sit upright unassisted) and retro-orbital pain were significantly more frequent among participants who later developed chronic manifestations; the former of which has not previously been considered as a predictor of chronic illness (Table 1). Viral load at presentation was not identified as a predictor of chronic chikungunya.

### Chronic manifestations of chikungunya

At D90, across the entire cohort, long-term manifestations were observed in 49/100 participants, of whom 38/49 (78%) met the study definition of chronic chikungunya (the presence of either arthralgia or myalgia). The remaining patients with long-term manifestations who did not meet the chronic chikungunya definition presented with rash (1/11, 9%), weakness (8/11, 73%) or altered vision (3/11, 27%) (Figure 2).

**Figure 2:**
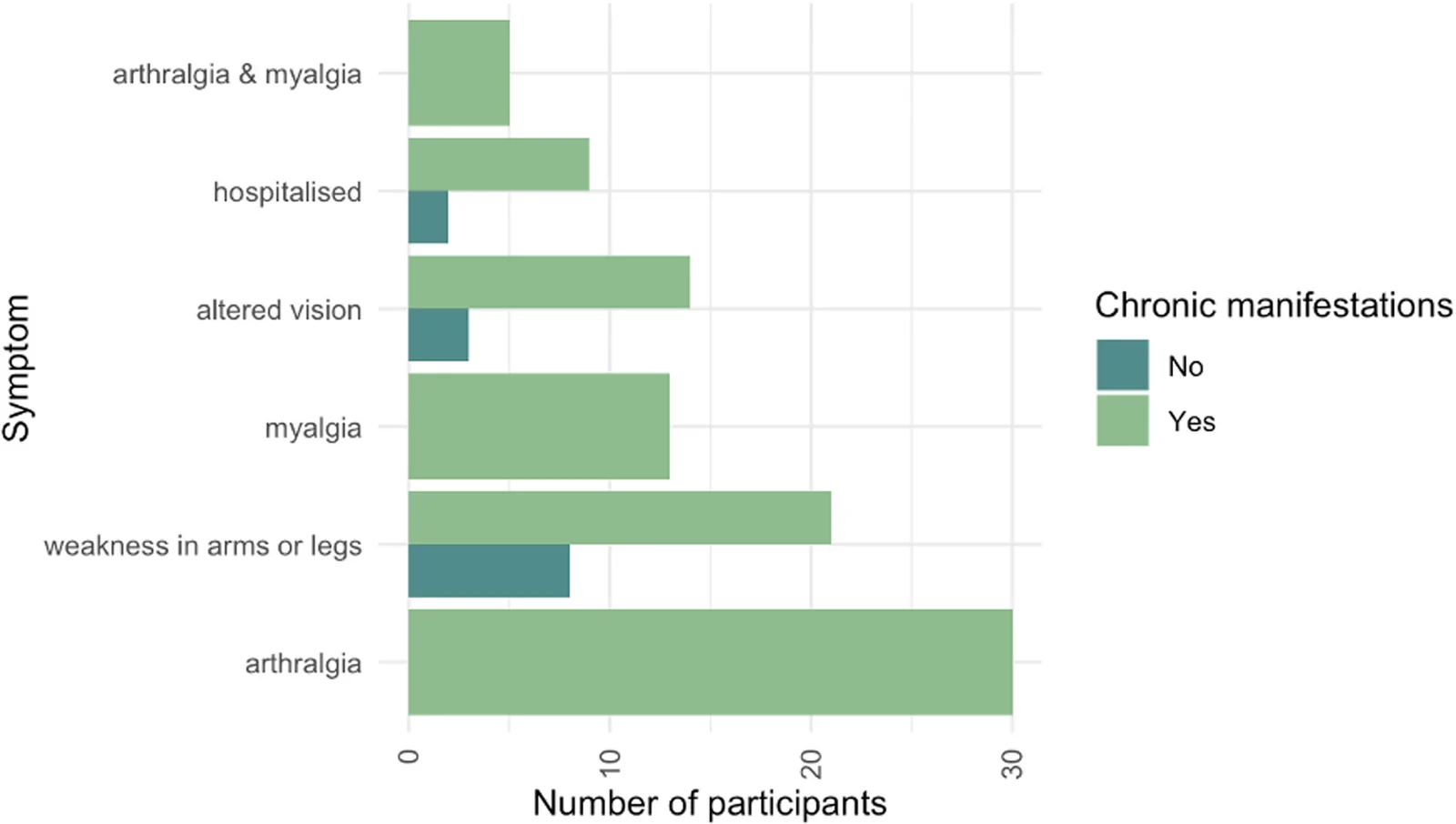
Frequency of long-term manifestations present at D90 among the 49 participants with ongoing signs and symptoms.

Of the participants with chronic chikungunya, arthralgia was reported by 30/38 (79%) participants, myalgia was reported by 13/38 (34%) participants, and 5/38 (13%) participants reported the presence of both symptoms (Figure 2; Supplementary material, Table S3). Arthralgia was reported to be symmetrical in 25 (83%) participants with chronic chikungunya and most frequently reported in ankles (37%), knees (32%) and shoulders (21%). All but one participant that presented with arthralgia on D90 reported arthralgia during the acute phase (Supplementary material, Table S4).

Participants with chronic chikungunya were more likely to report at D90 altered vision or hearing, weakness, and prior hospitalization (for any reason from initial presentation) compared with those who had fully recovered. In the multivariable logistic regression analysis, at D90 altered vision or hearing (OR 7.44, 95% CI 1.83-38.70), hospitalization (OR 10.59, 95% CI 2.05-81.66), and weakness (OR 7.67, 95% CI 2.66-23.97) were strongly associated with chronic disease status.

In a bivariable analysis, age, female sex, prostration, and retro-orbital pain at enrolment were associated with chronic chikungunya (Table 1). However, in multivariable logistic regression analysis, female sex and age were independently associated with increased odds of chronic chikungunya (female sex: adjusted OR 2.56, 95% CI 1.02-6.25, *P* = 0.045; age: adjusted OR 1.04, 95% CI 1.01-1.07, *P* = 0.024), and prostration and retro-orbital pain showed a trend towards association (prostration: adjusted OR 2.49, 95% CI 0.99-6.24, *P* = 0.053; retro-orbital pain: adjusted OR 2.45, 95% CI 0.98-6.11, *P* = 0.057).

### Quality of life

At D90, of the entire patient population, 77 (77%) participants reported being able to perform daily activities without difficulty, of whom 21 (27%) had chronic chikungunya (Figure 3). Twenty-one participants (21%) reported that they could perform daily activities with some limitations, of whom a higher proportion (71%) had chronic chikungunya. Two (2%) participants reported that could not perform daily activities, both of whom had chronic chikungunya.

**Figure 3:**
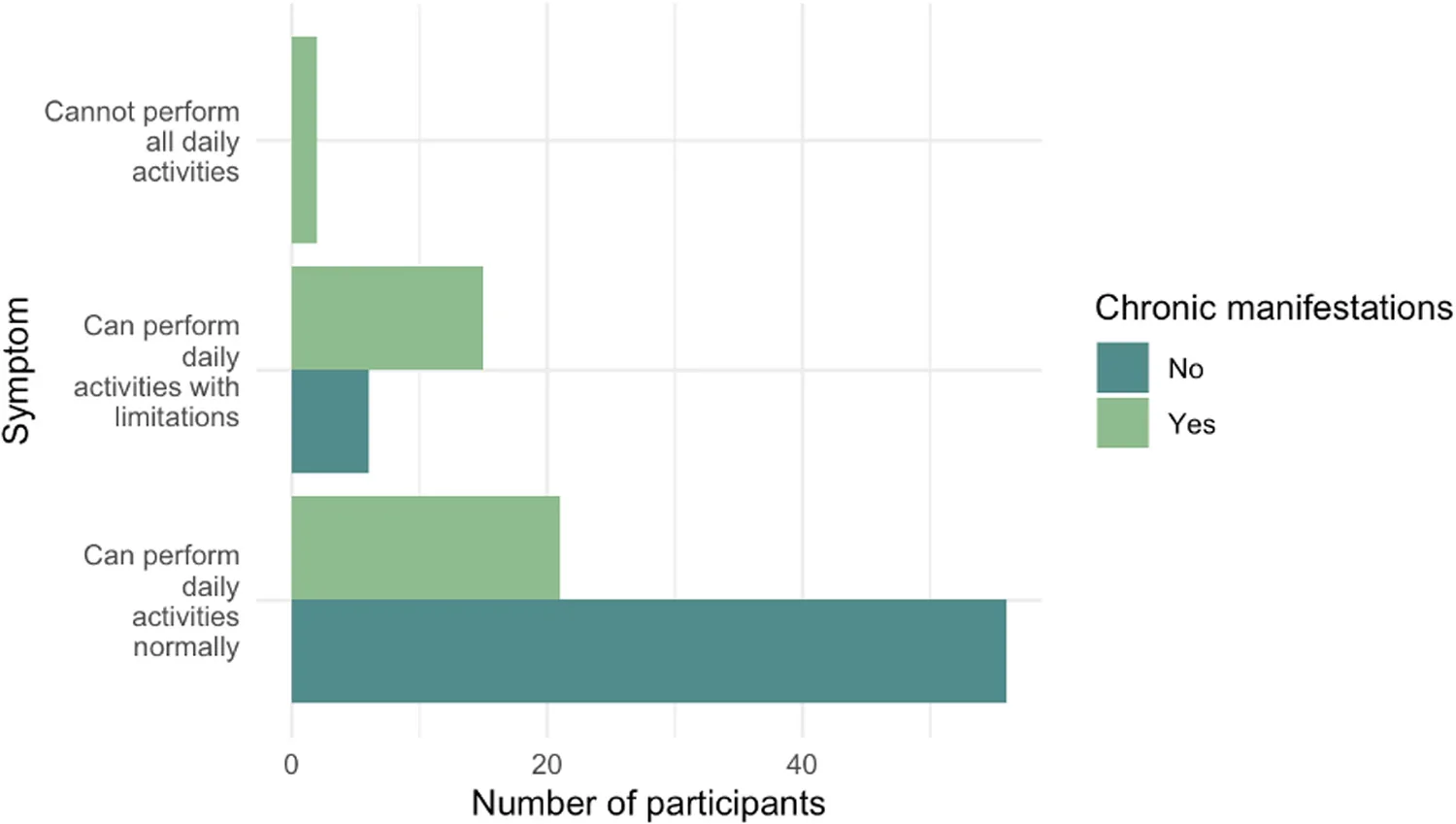
Health-related quality of life at D90 among participants with and without chronic manifestations of chikungunya.

One third of participants reported pain at the point of the D90 visit, of whom 30 (91%) had chronic chikungunya. When asked about the level of pain experienced during the telephone call, 12 participants (36%) reported a pain score of five and above (Supplementary material, Figure S2). When asked about the impact of pain on their daily lives, approximately one quarter reported a substantial impact on their ability to walk or work, their mood and joy of living, their relationships and their sleep (Supplementary material, Figure S3).

Overall higher proportions of participants with chronic chikungunya reported poorer quality of life than those without (Supplementary Figure S4). More participants living with chronic chikungunya reported that their physical and mental health resulted in moderate to substantial limitations when undertaking daily activities (including work) and noted changes in the type of activities they could perform.

## Discussion

In this prospective cohort study, we provide a detailed clinical characterization of chikungunya virus infection from 100 of the 134 adults with RT-PCR confirmed chikungunya enrolled in the study presenting to an emergency department in Rio de Janeiro, with longitudinal follow-up extending to 90 days after enrolment. Our findings confirm the substantial burden of chronic manifestations following chikungunya and add important insight into symptom profiles during the acute phase, potential early predictors of chronic disease, and the long-term impact on quality of life.

The acute clinical presentation observed in this cohort was typical of chikungunya virus infection, with fever, arthralgia, myalgia, and headache reported by the majority of patients. Approximately one third of participants reported chronic manifestations at 90 days post-enrolment, lower than reported in recent systematic reviews describing persistent symptoms [13,14]. This difference may reflect methodological and population differences between studies. First, chronic chikungunya was defined in our study as the presence of arthralgia or musculoskeletal pain at the D90 follow-up assessment, rather than continuous or recurrent symptoms over a defined period, which may have resulted in a lower estimated prevalence. Second, participants were recruited from the emergency department of a private hospital, where differences in healthcare access, socioeconomic characteristics and healthcare-seeking behavior may influence both symptom management and follow-up. Finally, our cohort consisted exclusively of laboratory-confirmed cases recruited during the acute phase of illness, whereas previous studies have included more heterogeneous populations with varying case definitions and follow-up intervals. These factors should be considered when comparing prevalence estimates across studies.

Two clinical scoring systems, SHERA and CCA, have previously been developed to identify predictors of chronic chikungunya [22,23]. SHERA highlights the role of oedema, age >26 years, retro-orbital pain, female sex, and hypertension [22], while the CCA score emphasizes female sex, comparably older age (>40 years), extensive joint involvement (≥8 arthralgia sites), and self-reported arthralgia [23]. In our cohort, the exploratory application of the SHERA score yielded a positive predictive value (PPV) of 0.60 for chronic chikungunya, indicating limited predictive performance in our setting. Calculation of the CCA score was not feasible due to the absence of key required variables, precluding direct comparison of the two predictive tools.

In our cohort, female sex and age were independently associated with increased odds of chronic chikungunya. Both are consistent with existing SHERA and CCA scoring systems. Prostration and retro-orbital pain at enrolment demonstrated a trend towards association but did not reach conventional statistical significance. In contrast, pre-existing hypertension, while common in this cohort, was not significantly associated with chronic outcomes, suggesting that cardiovascular comorbidity alone may not be a major determinant of chronic chikungunya manifestations.

Although female sex and age were independently associated with chronic chikungunya, and retro-orbital pain and prostration showed a trend towards association, in the multivariable analysis, the limited number of outcome events in our cohort precluded robust development of a new prognostic model. Our findings should therefore be interpreted as hypothesis-generating and warrant validation in larger prospective cohorts.

Importantly, our study adds to the limited prospective data on the long-term impact of chikungunya on quality of life in Brazil. Although more than half of participants reported being able to perform daily activities without difficulty 90 days after diagnosis, a substantial proportion continued to experience clinically meaningful impairment, with persistent symptoms adversely affecting individual-level quality of life. Approximately one third of participants reported moderate to severe interference of pain with mood, work, and sleep, and over one quarter reported similar interference with general activities and walking ability. These findings underscore the heterogeneous nature of recovery following chikungunya infection and highlight that even in the absence of severe disability, persistent pain can significantly affect multiple domains of daily functioning and supports the need for long-term follow-up and multidisciplinary management strategies.

## Limitations

This study has several strengths, including its prospective design, laboratory-confirmed diagnosis, and extended follow-up. However, several limitations should be considered. Loss to follow-up at D90, follow-up limited to 90 days post-enrolment, and reliance on self-reported symptoms during telephone interviews may have introduced attrition and recall bias.

The study was conducted in the emergency department of a single private hospital in Rio de Janeiro, where participants were likely to have greater access to healthcare and follow-up than the general population. This may have increased the likelihood of seeking care for persistent symptoms and completing scheduled study visits. As patterns of healthcare utilization in Brazil vary according to socioeconomic status and use of private vs public health services, the study population may not be generalizable to all patients with chikungunya, particularly those relying exclusively on the public healthcare system [24].

Chronic chikungunya was defined according to the presence of arthralgia or musculoskeletal pain at the D90 follow-up assessment rather than by continuous symptom persistence from illness onset. As enrolment did not occur at a standardized interval following symptom onset, the timing of the D90 assessment varied slightly between participants. Furthermore, symptom trajectory between enrolment and follow-up was not systematically documented; therefore, we were unable to distinguish between continuous, intermittent, or recurrent symptoms. This cross-sectional definition may have resulted in some misclassification and should be considered when interpreting the reported prevalence of chronic chikungunya.

## Conclusion

Overall, these findings suggest that clinical features at initial presentation may help identify patients at increased risk of chronic disease, though validation in larger cohorts is warranted. Our results also highlight the substantial and persistent impact of chikungunya virus infection beyond the acute phase, with a significant proportion of patients experiencing chronic manifestations and impaired quality of life at 90 days post-enrolment. These findings reinforce the need for improved recognition of chronic chikungunya, early identification of patients at risk of persistent symptoms, effective communication management towards the patient, and the integration of long-term care strategies into arboviral disease management in endemic regions.

## Data availability

Access to de-identified data will be made available upon reasonable request to the corresponding author.

## Funding

This study was funded by Charité – Universitätsmedizin Berlin (Award number: 8040801916).

## CRediT authorship contribution statement

**Josephine Bourner:** Writing – review & editing, Writing – original draft, Visualization, Formal analysis. **Alessandra Dantas do Nascimento:** Investigation. **Wim Alexander Fleischmann:** Writing – original draft, Formal analysis. **Jeni Stolow:** Writing – review & editing. **Leonardo L.S. Bastos:** Writing – review & editing. **Grazielle Viana Ramos:** Project administration. **Patricia T. Bozza:** Writing – review & editing, Investigation. **Thiago L. Moreno Souza:** Writing – review & editing, Investigation. **Jan Felix Drexler:** Supervision. **Jose Cerbino Neto:** Supervision. **Fernando A. Bozza:** Supervision.

## Declaration of competing interest

The authors declare that they have no known competing financial interests or personal relationships that could have appeared to influence the work reported in this paper.

## Appendix Supplementary materials

Image, application 1
