# Supplementary material for: Characterization of the clinical forms of chronic chikungunya: an observational cohort study in Rio de Janeiro
Source: Int J Infect Dis. 2026 Oct;171:None. doi: 10.1016/j.ijid.2026.109050 (PMC13615952; doi:10.1016/j.ijid.2026.109050)
Supplement: Supplementary file 1 [file mmc1.docx]

**Supplementary material**

**Text S1. Methodology for molecular diagnosis**

RNA extraction

Viral genomic RNA was isolated from the specimens using the QIAamp Viral RNA Mini Kit (Qiagen®, Düsseldorf, Germany) following the protocol provided by the manufacturer. The final elution yielded 60 µL of purified RNA, which was subsequently stored for real-time RT-PCR analysis. Negative extraction controls were processed alongside the samples throughout the entire procedure to monitor for potential cross-contamination.

Real-time RT-PCR analysis

The real-time RT-PCR amplifications were conducted in a final reaction volume of 25 µL using the QuantiTect/QuantiNova Probe RT-PCR Kit (Qiagen®). Each reaction mixture contained 5 µL of the extracted RNA template, 1 µM of each forward and reverse primer, and 0.2 µM of the respective probe (sequences detailed in Table S1). The thermal cycling conditions consisted of a 30-min reverse transcription step at 50 °C, followed by an initial denaturation phase at 95 °C for 15 min. This was succeeded by 50 amplification cycles of denaturation (94 °C for 15 s) and annealing (55 °C for 35 s). Samples demonstrating a sigmoidal amplification curve with a cycle threshold (Ct) below 40 were classified as positive.

Samples were screened for the four DENV serotypes, CHIKV, and ZIKV. Rigorous quality assurance was maintained by including several controls in each run: extraction mock-controls, non-template RT-PCR controls, specific positive controls for each target pathogen, and human 18S rRNA (ThermoFisher, Waltham, Massachusetts, USA) as an internal reference for specimen integrity.

**Table S1. Description of published primer/ probe sets used in qPCR**

| Virus target | Reference | Primer/ probe | Genome position | Sequence (5’-3’) |
| --- | --- | --- | --- | --- |
| ZIKV | Lanciotti *et al*, 2008 (PMID: 18680646) | Primer F  Primer R  Probe | 1086-1102  1162-1139  1107-1137 | CCG CTG CCC AAC ACA AG  CCA CTA ACG TTC TTT TGC AGA CAT  FAM/ AGC CTA CCT TGA CAA GCA GTC AGA CAC TCA A /BHQ1 |
| CHIKV | Lanciotti *et al*, 2007 (PMID: 17553261) | Primer F  Primer R  Probe | 874-894  961-942  899-923 | AAA GGG CAA ACT CAG CTT CAC  GCC TGG GCT CAT CGT TAT TC  FAM/ CGC TGT GAT ACA GTG GTT TCG TGT G/BHQ1 |
| DENV1 | Santiago *et al*, 2013 (PMID: 23875046) | Primer F  Primer R  Probe | 8936-8955  9023-9047  8961-8979 | CAA AAG GAA GTC GYG CAA TA  CTG AGT GAA TTC TCT CTG CTR AAC  FAM/CAT GTG GYT GGG AGC RCG C/BHQ1 |
| DENV2 | Santiago *et al*, 2013 (PMID: 23875046) | Primer F  Primer R  Probe | 1426-1447  1482-1504  1454-1480 | CAG GCT ATG GCA CYG TCA CGA T  CCA TYT GCA GCA RCA CCA TCT C  VIC/CTC YCC RAG AAC GGG CCT CGA CTT CAA/BHQ1 |
| DENV3 | Santiago *et al*, 2013 (PMID: 23875046) | Primer F  Primer R  Probe | 701-720  749-775  722-747 | GGA CTR GAC ACA CGC ACC CA  CAT GTC TCT ACC TTC TCG ACT TGY CT  FAM/ACC TGG ATG TCG GCT GAA GGA GCY TG/BHQ1 |
| DENV4 | Santiago *et al*, 2013 (PMID: 23875046) | Primer F  Primer R  Probe | 884-904  953-973  939-965 | TTG TCC TAA TGA TGC TRG TCG  TCC ACC YGA GAC TCC TTC CA  FAM/TYC CTA CYC CTA CGC ATC GCA TTC CG/BHQ1 |

**Table S2. Completeness of key study variables.**

| **Variable** | **Available**  **n/100 (%)** | **Missing**  **n/100 (%)** |
| --- | --- | --- |
| ***Demographic information*** | | |
| Sex | 100 (100%) | 0 (0%) |
| Age | 97 (97%) | 3 (3%) |
| Pregnancy | 100 (100%) | 0 (0%) |
| Hypertension | 100 (100%) | 0 (0%) |
| Diabetes | 100 (100%) | 0 (0%) |
| COPD | 100 (100%) | 0 (0%) |
| Cardiopathy | 100 (100%) | 0 (0%) |
| ***Baseline signs and symptoms*** | | |
| Time between symptom onset and presentation | 95 (95%) | 5 (5%) |
| Viral load | 83 (83%) | 17 (17%) |
| Fever | 100 (100%) | 0 (0%) |
| Arthralgia | 100 (100%) | 0 (0%) |
| Myalgia | 100 (100%) | 0 (0%) |
| Headache | 100 (100%) | 0 (0%) |
| Prostration | 100 (100%) | 0 (0%) |
| Retro-orbital pain | 100 (100%) | 0 (0%) |
| Rash | 95 (95%) | 5 (5%) |
| Nausea and vomiting | 100 (100%) | 0 (0%) |
| Limb oedema | 100 (100%) | 0 (0%) |
| Weakness | 100 (100%) | 0 (0%) |
| Tiredness | 100 (100%) | 0 (0%) |
| Conjunctivitis | 100 (100%) | 0 (0%) |
| Difficulty swallowing | 100 (100%) | 0 (0%) |
| Arthritis | 52 (52%) | 48 (48%) |
| ***Long-term manifestations*** | | |
| Arthralgia | 100 (100%) | 0 (0%) |
| Myalgia | 100 (100%) | 0 (0%) |
| Weakness in arms or legs | 100 (100%) | 0 (0%) |
| Altered vision | 100 (100%) | 0 (0%) |
| Hospitalisation | 100 (100%) | 0 (0%) |
| ***Quality of life questionnaire*** | 100 (100%) | 0 (0%) |

**Table S3. Comparison of baseline characteristics of RT-PCR positive participants who completed follow-up (n = 100) and those who did not complete follow-up (n = 34)**

|  | **Participants who completed follow-up**  **(n=100)** | **Participants who did not complete follow-up**  **(n = 34)** |
| --- | --- | --- |
| ***Demographic characteristics*** | | |
| Sex (female:male) | 50:50 | 20:14 |
| Age (years), median (range) | 44 (19-87) | 41 (18-94) |
| Pregnancy (%) | 2 (2%) | 0 (0%) |
| ***Comorbidities*** | | |
| Hypertension (%) | 25 (25%) | 8 (24%) |
| Diabetes (%) | 8 (8%) | 3 (9%) |
| COPD (%) | 1 (1%) | 1 (3%) |
| Cardiopathy (%) | 1 (1%) | 0 (0%) |
| Multiple comorbidities | 7 (7%) | 2 (6%) |
| ***Acute phase signs and symptoms*** | | |
| Time between symptom onset and presentation (days), median (range) | 2 (1-7) | 2 (1-7) |
| Viral load (log10 copies/mL), median (range) | 1.8 (-1.09 4.33) | 2.2 (0.4-3.7) |
| Fever, n (%) | 98 (98%) | 32 (94%) |
| Arthralgia, n (%) | 94 (94%) | 31 (91%) |
| Myalgia, n (%) | 77 (77%) | 21 (62%) |
| Headache, n (%) | 75 (75%) | 25 (74%) |
| Prostration, n (%) | 47 (47%) | 16 (47%) |
| Retro-orbital pain, n (%) | 41 (41%) | 13 (38%) |
| Rash, n (%) | 35 (35%) | 8 (24%) |
| Nausea and vomiting, n (%) | 31 (31%) | 11 (32%) |
| Limb oedema, n (%) | 24 (24%) | 8 (24%) |
| Weakness, n (%) | 20 (20%) | 16 (47%) |
| Tiredness, n (%) | 4 (4%) | 4 (12%) |
| Conjunctivitis, n (%) | 2 (2%) | 1 (3%) |
| Difficulty swallowing, n (%) | 2 (2%) | 0 (0%) |
| Arthritis, n (%) | 1 (1%) | 1 (3%) |

**Figure S1. Signs and symptoms present during the acute phase.**

**Table S4. Chronic manifestations of chikungunya.**

|  | **All participants**  **(n=100)** | **With chronic manifestations**  **(n=38)** | **Without chronic manifestations**  **(n=62)** | **p-value** |
| --- | --- | --- | --- | --- |
| Musculoskeletal symptoms (n = 38) | | | | |
| Arthralgia, n (%) | 30 (30%) | 30 (79%) | 0 |  |
| Muscle pain, n (%) | 13 (13%) | 13 (34%) | 0 |  |
| Arthralgia and muscle pain, n (%) | 5 (5%) | 5 (13%) | 0 |  |
| Other symptoms | | | | |
| **Weakness in arms and legs, n (%)** | **29 (29%)** | **21 (55%)** | **8 (13%)** | **<0.0001** |
| **Altered vision or hearing, n (%)** | **17 (17%)** | **14 (36%)** | **3 (4%)** | **<0.0001** |
| **Hospitalisation during study period, n (%)** | **11 (11%)** | **9 (23%)** | **2 (3%)** | **0.0044** |

**Table S5. Number of participants with chronic chikungunya reporting arthralgia (N = 30) according to location.**

| **Location of arthralgia** | **N participants reporting arthralgia**  **(N = 30)** |
| --- | --- |
| **Multiple locations** | **14 (46%)** |
| **Neck** | **3 (10%)** |
| **Shoulders** | **8 (27%)** |
| **Lower back** | **1 (3%)** |
| **Upper limbs** | **12 (40%)** |
| Elbows | 4 (13%) |
| Wrists | 7 (23%) |
| Hands | 5 (17%) |
| Fingers | 2 (7%) |
| **Lower limbs** | **20 (67%)** |
| Knees | 11 (37%) |
| Ankles | 14 (46%) |
| Feet | 4 (13%) |

**Figure S2 – Pain scores (scale of 1 to 10) reported at the point of the D90 visit and within the 7 days before the D90 visit among patients with chronic chikungunya (N = 38)**

**Figure S3 – Impact of pain (on a scale of 1 to 10) on quality of life reported at the D90 telephone interview among participants reporting pain (N = 30)**

**Figure S4 – Impression of the impact of chikungunya on quality of life across entire cohort (N = 100)**

**In the last four weeks…**

**… has your health prevented you undertaking moderate activities?**

**…how would you describe your general health?**

**… has your mental health prevented you undertaking your usual activities?**

**… has your health prevented you from climbing stairs?**

**In the last four weeks…**

**… have you paid less attention to your work or activities due to your emotional state?**

**… has your physical health changed the type of work or activities you undertake?**

**… have you felt calm or peaceful?**

**… how much has pain interfered with your usual activities?**

**In the last four weeks…**

**… have you felt hopeless or depressed?**

**… have you felt happy and cheerful?**

**… has your physical health or emotional state interfered with your social life?**
